# Comprehensive studies reveal physiological and genetic diversity in traditional rice cultivars for UV-B sensitivity

**DOI:** 10.1038/s41598-024-64134-0

**Published:** 2024-06-07

**Authors:** Preetam Kumar Senapati, Ekamber Kariali, Kuntala Kisan, Binod Bihari Sahu, Arya Kumar Dibyananda Naik, Debabrata Panda, Santanu Kumar Tripathy, Sanjukta Mohapatra, Pravat Kumar Mohapatra

**Affiliations:** 1 School of Life Sciences, Sambalpur University, Sambalpur, 768019 India; 2 Department of Life Science, National Institute of Technology, Rourkela, 769008 India; 3 Department of Biodiversity and Conservation, Central University of Odisha, Koraput, 763004 India; 4 Odisha University of Agriculture and Technology, Bhubaneswar, 751003 India; 5 Regional Research and Technology Transfer Station, Chipilima, 768025 India

**Keywords:** Anthocyanin, Flavonoid, UV-B stress-sensitive genes, Pubescent hairs, Rice, Photosynthetic parameters, Physiology, Plant sciences

## Abstract

Acclimation to crop niches for thousands of years has made indigenous rice cultivars better suited for stress-prone environments. Still, their response to UV-B resiliency is unknown. 38 rice landraces were grown in cemented pots in a randomised block design with three replicates under open field conditions in Sambalpur University in the wet season of 2022. Half of the plants in each of the cultivars were administered UV-B radiation at the panicle emergence stage in an adjustable UV-B chamber permitting sunlight, and the effects of the stress on various morpho-physiological features, such as spikelet sterility, flag leaf photosynthetic and flavonoid pigment contents, and lipid peroxidation activities, were estimated for calibration of stress resistance. The experiment identified Swarnaprabha and Lalkain as the most sensitive and resilient to stress respectively, and the differential response between them was further revealed in the expression of genes related to UV-B sensitivity. Subject to the stress, Swarnaprabha exhibited symptoms of injuries, like leaf burns, and a higher loss of various photosynthetic parameters, such as pigment contents, SPAD and Fv/Fm, ETR and qP values, while NPQ increased only in Lalkain. Exposure to UV-B increased the total phenolic and flavonoid contents in Lalkain while depressing them in Swarnaprabha. Such an effect amounted to a higher release of fluorescent energy in the latter. The levels of expression of gene families controlling flavonoid activation and UV-B signal transduction, such as *OsWRKY*,* OsUGT*,* OsRLCK*,* OsBZIP*, *OsGLP*, and *CPD photolyase* were similar in both the cultivars in the control condition. However, exposure to UV-B stress overexpressed them in resilient cultivars only. The magnitude of expression of the genes and the impact of the stress on photosynthetic parameters, phenolic compounds and pubescent hair structure at the panicle emergence stage could be valid indicators among indigenous rice for UV-B tolerance.

## Introduction

Rice, similar to other plants uses light energy for photosynthesis and sustenance, and cannot avoid exposure to enhanced levels of UV radiation in the agro-ecosystem. While UV-A radiation is beneficial to plants, all living organisms are at risk of exposition in the UV-B (280–315 nm) range. In recent years, global climate change has depleted the stratospheric ozone layer leading to a significant increase in exposure of the biosphere to unfiltered UV-B radiation, which is harmful to plants and animals. Akin to other organisms, the deleterious UV-B radiation triggers injuries in rice plants by altering changes in several morphological and physiological features. Rice is a prime food crop in the tropical regions of the world. Compared to this region, the incidence of UV-B radiation causing yield loss in the temperate climate is much lower^1^. Being located in a low ozone belt, UV-B stress damages nearly 50% of the crops in India, because they receive high levels of unfiltered radiation^2,3^. According to Bhattacharya^4^, climate change-induced stratospheric ozone deficiency, as much as 4% over the tropical Asian rice belt impacts rice crops as the most delimiting abiotic factor. As a result, several phenotypic abnormalities like stunting, leaf blotching, spikelet number alteration, and grain number reduction appear in the crop as symptoms of injury on productive tillers^5^. However, the impact of underlying mechanisms governing physiological and biochemical changes of UV-B stress although tantamount to significant depression of grain yield and plant phenology is seldom investigated. A comparison of UV-B sensitivity in 13 elite high-yielding rice cultivars revealed that the deleterious radiation was harmful in sensitive cultivars by promoting oxidative stress consequently resulting in a rise of malondialdehyde content (MDA) and damage to the leaf photosynthetic system, compromising the free radical scavenging process^6^. In the flag leaf of *japonica* rice commonly grown in Yuan yang, Japan, the incidence of enhanced UV-B stress increased contents of brassinolide and gibberellin, but reduced auxin content; the cellulose content of the pulvinus decreased making the leaf angle wider than before^7^.

Because high-yielding rice cultivars have exclusively contributed to increased food grain production over the last 50 years, they have received priority in screening for physiological and biochemical responses to UV-B stress. In a new paradigm for rice agriculture in the International Rice Research Institute, Philippines to meet the global challenge program on bio-fortification and alleviate the nutritional deficiencies in rice consumers^8^, attention was focused on local native unimproved traditional rice cultivation. In reality, many rice consumers prefer indigenous traditional rice over high-yielding rice because of their taste, aroma, texture, and storehouse for nutrients and bioactive compounds for therapeutic use^9,10^. Also, some of the traditional rice genotypes contain high levels of phenolic compounds including polyphenols, anthocyanin, and flavonoids^10^ but their utility for UV-B radiation resiliency has not been tested hitherto. The traditional rice cultivars are ecotypes domesticated to a particular niche having evolved there for many generations to adapt highly to local physicochemical and biological stresses^11^ because of habitat-specific spontaneous mutation. However, there is scant knowledge of the identity of a descriptor for UV-B resistance. In pursuance, the expression of various gene families for flavonoid activation and UV-B signal transduction could elucidate the mode of sensitivity to stress by rice plants. *CPD* (Cyclobutane Pyridine Dimer) *Photolyase* expression indicates UV-B damage repair mechanisms^12^. *OsRLCK160* expression contributes to flavonoid accumulation and UV-B tolerance by regulating *OsbZIP48*^13^. A flavonoid 7-O-glycosyltransferase (*OsUGT706C2*) gene has a role in modulating flavonol (kaempferol), and flavone (luteolin and chrysoeriol) metabolism^14^. Rice germin-like protein *OsGLP1* participates in acclimation to UV-B radiation^15^ and the overexpression of genes *WRKY89* and *WRKY109* enhances UV-B tolerance and disease resistance in rice plants^16^.

It is assumed that UV-B stress emerged late in the biosphere because of the congruence between conservation measures and the generation of industrial pollutants. Although adapted to other forms of abiotic stress centuries ago in their natural habitats, traditional rice might not have experienced this recently emerged stress. Sensitivity to UV-B stress varies widely among rice cultivars^17^; a poor cultivar choice on the part of the farmer therefore is expected to be counter-productive for harnessing optimum yield. In this context, the objective of the present dispensation has been to screen some of these traditional genotypes available locally for UV-B stress resiliency and make appropriate recommendations.

## Results

### Cultivar screening for UV-B sensitivity

Depending on the response to UV-B radiation, all 38 cultivars were classified into four distinct groups (Table 1). The first group had two highly tolerant cultivars, namely Lalkain and Meghadambaru. UV-B stress impacted the % of fertile grain number in them, but the effect was low in this group compared to the other three groups. Similarly, % of the total chlorophyll contents of the flag leaf declined marginally under the harmful radiation. Conversely, % of lipid peroxidation, and anthocyanin and flavonoid concentration increased in the flag leaf under harmful radiation. According to these five morpho-physiological descriptors of the UV-B stress effects, the rest of the 36 cultivars were separated into 3 groups. 19 cultivars belonged to the moderately resistant class, whereas 15 cultivars were sensitive and the residual two cultivars were susceptible to the stress.

**Table 1:** Categorization of 38 rice cultivars into four groups (Highly Resistant, resistant, sensitive and highly sensitive)  ± Values indicates SD values (n = 3).

	Rice cultivars	% of total chlorophyll Increase(+) or Decrease(−)	% of lipid-peroxidation Increase(+) or Decrease(−)	% of anthocyanin Increase(+) or Decrease(−)	% of flavonoid Increase(+) or Decrease(−)	% of fertile grain number Increase(+) or Decrease(−)
Highly resistant	Lalkain	− 02.86 ± 0.22%	+ 05.92 ± 0.25%	+ 39.50 ± 0.07%	+ 12.86 ± 0.09%	− 04.71 ± 0.23
	Meghadambaru	− 04.08 ± 0.02%	+ 06.62 ± 0.16%	+ 28.20 ± 0.07%	+ 10.60 ± 0.08%	− 06.82 ± 0.14
Resistant	Lalgudi	− 06.54 ± 0.03%	+ 12.84 ± 0.11%	+ 09.37 ± 0.34%	+ 02.46 ± 0.05%	− 10.89 ± 0.28
	Kalamalifuli	− 09.17 ± 0.12%	+ 11.61 ± 0.12%	+ 03.44 ± 0.11%	+ 03.12 ± 0.06%	− 10.85 ± 0.45
	Manipuri black	− 09.91 ± 0.05%	+ 10.65 ± 0.07%	+ 03.33 ± 0.05%	+ 02.12 ± 0.06%	− 11.31 ± 0.44
	Pakhiraj	− 07.28 ± 0.03%	+ 11.56 ± 0.06%	+ 06.45 ± 0.05%	+ 05.61 ± 0.11%	− 13.47 ± 0.43
	Karnaful	− 08.92 ± 0.03%	+ 12.39 ± 0.09%	+ 09.09 ± 0.17%	+ 07.40 ± 0.04%	− 14.78 ± 0.53
	Laha	− 08.19 ± 0.08%	+ 14.74 ± 0.06%	+ 11.42 ± 0.08%	+ 06.02 ± 0.13%	− 15.12 ± 0.36
	Budi	− 09.12 ± 0.09%	+ 11.50 ± 0.12%	+ 03.12 ± 0.11%	+ 02.50 ± 0.05%	− 15.99 ± 0.36
	Khara	− 07.56 ± 0.06%	+ 15.45 ± 0.05%	+ 12.65 ± 0.10%	+ 06.38 ± 0.06%	− 08.23 ± 0.52
	Kalinagin	− 09.78 ± 0.07%	+ 15.02 ± 0.14%	+ 07.89 ± 0.19%	+ 04.00 ± 0.13%	− 13.55 ± 0.50
	Baspatri	− 07.62 ± 0.07%	+ 14.47 ± 0.08%	+ 12.12 ± 0.08%	+ 07.69 ± 0.10%	− 14.76 ± 0.55
	Kalasu	− 09.54 ± 0.04%	+ 15.50 ± 0.09%	+ 03.44 ± 0.10%	+ 01.13 ± 0.05%	− 14.13 ± 0.37
	Burma black	− 08.73 ± 0.05%	+ 13.54 ± 0.05%	+ 05.40 ± 0.09%	+ 05.15 ± 0.09%	− 15.28 ± 0.45
	Karni	− 08.77 ± 0.07%	+ 15.48 ± 0.04%	+ 10.00 ± 0.13%	+ 04.65 ± 0.05%	− 13.17 ± 0.20
	Pahadijeera	− 07.55 ± 0.05%	+ 15.82 ± 0.21%	+ 10.81 ± 0.08%	+ 03.48 ± 0.07%	− 09.38 ± 0.41
	Kalia	− 09.17 ± 0.09%	+ 14.41 ± 0.14%	+ 08.82 ± 0.09%	+ 07.52 ± 0.13%	− 09.77 ± 0.45
	Kalajeera	− 08.69 ± 0.04%	+ 15.88 ± 0.14%	+ 02.70 ± 0.05%	+ 01.29 ± 0.07%	− 10.01 ± 0.35
	Aithira	− 09.20 ± 0.08%	+ 09.43 ± 0.11%	+ 08.82 ± 0.09%	+ 06.66 ± 0.13%	− 10.29 ± 0.78
	Kalabati	− 06.75 ± 0.04%	+ 14.97 ± 0.21%	+ 16.21 ± 0.07%	+ 07.31 ± 0.05%	− 12.07 ± 0.45
	kanchan	− 07.40 ± 0.06%	+ 12.07 ± 0.22%	+ 05.88 ± 0.08%	+ 03.37 ± 0.06%	− 10.43 ± 0.49
Sensitive	Barsa	− 17.05 ± 0.52%	+ 20.31 ± 0.06%	− 06.89 ± 0.05%	− 02.38 ± 0.07%	− 24.08 ± 0.59
	Kusumchupa	− 17.30 ± 0.17%	+ 19.64 ± 0.07%	− 10.71 ± 0.09%	− 03.65 ± 0.06%	− 24.32 ± 0.32
	Deradun	− 20.97 ± 0.27%	+ 19.88 ± 0.22%	− 08.00 ± 0.08%	− 01.29 ± 0.07%	− 25.55 ± 0.56
	Bharatsanda	− 19.81 ± 0.14%	+ 21.52 ± 0.29%	− 16.66 ± 0.07%	− 05.47 ± 0.13%	− 25.99 ± 0.58
	Samanta	− 19.66 ± 0.07%	+ 21.50 ± 0.11%	− 11.53 ± 0.07%	− 03.84 ± 0.06%	− 21.06 ± 0.86
	Kusuma	− 22.58 ± 0.10%	+ 21.90 ± 0.22%	− 16.00 ± 0.09%	− 02.40 ± 0.08%	− 23.14 ± 0.29
	Radhajugal	− 15.19 ± 0.18%	+ 19.79 ± 0.07%	− 08.00 ± 0.14%	− 04.70 ± 0.08%	− 23.95 ± 0.41
	Laisiring	− 18.40 ± 0.13%	+ 21.62 ± 0.07%	− 07.40 ± 0.13%	− 04.93 ± 0.15%	− 25.73 ± 0.30
	Lalpari	− 14.92 ± 0.25%	+ 20.12 ± 0.15%	− 12.00 ± 0.09%	− 03.65 ± 0.07%	− 21.87 ± 0.71
	Bagrijhuli	− 14.58 ± 0.09%	+ 19.78 ± 0.10%	− 14.81 ± 0.08%	− 08.00 ± 0.10%	− 20.29 ± 0.56
	Jhalkakiri	− 17.27 ± 0.07%	+ 20.12 ± 0.13%	− 06.89 ± 0.05%	− 03.65 ± 0.05%	− 23.52 ± 0.37
	Dhabjeera	− 20.78 ± 0.09%	+ 20.28 ± 0.08%	− 08.00 ± 0.04%	− 02.40 ± 0.05%	− 22.81 ± 0.34
	Debanna	− 21.37 ± 0.06%	+ 20.66 ± 0.08%	− 03.84 ± 0.11%	− 01.26 ± 0.07%	− 27.69 ± 0.55
	Mugudi	− 15.93 ± 0.18%	+ 20.63 ± 0.06%	− 12.50 ± 0.10%	− 02.66 ± 0.07%	− 27.87 ± 0.34
	Khudia	− 21.57 ± 0.18%	+ 20.79 ± 0.16%	− 12.50 ± 0.06%	− 01.31 ± 0.05%	− 22.96 ± 0.67
Highly sensitive	Swarnaprabha	− 40.47 ± 0.24%	+ 38.62 ± 0.09%	− 30.30 ± 0.15%	− 45.61 ± 0.17%	− 42.91 ± 0.54
	Sunakathi	− 32.96 ± 0.16%	+ 26.42 ± 0.04%	− 23.80 ± 0.13%	− 20.28 ± 0.08%	− 31.19 ± 0.49

### Effects of UV-B radiation on sensitive and tolerant cultivars

Lalkain (Highly resistant) and Swarnaprabha (highly sensitive) were chosen for an elegant run-off experiment for elucidation of the UV-B radiation stress effects precisely on the traditional rice.

#### Morphological features

**Figure 1:**
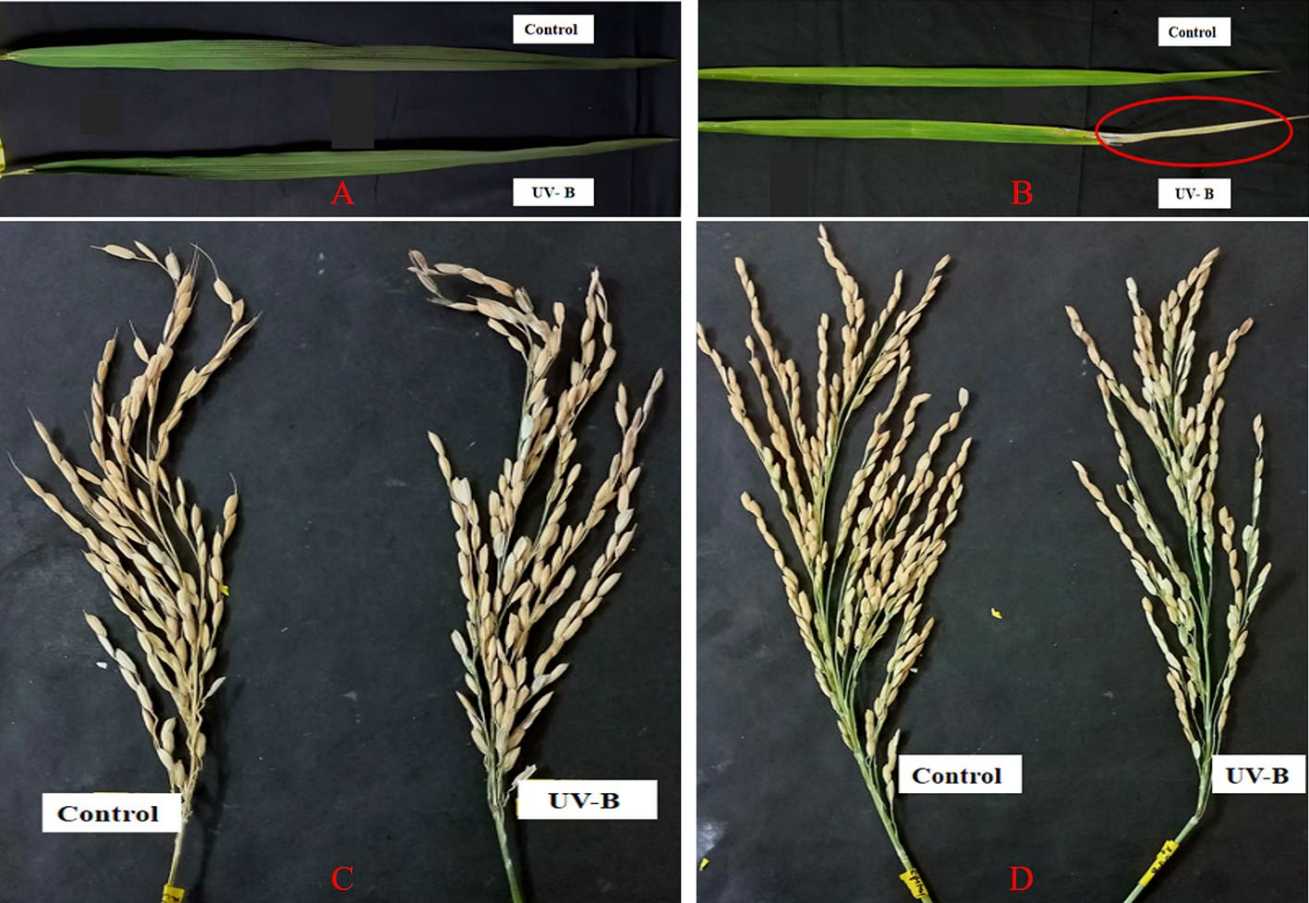
Effect of UV-B radiation on flag leaf and panicle size of rice genotypes *Lalkain* (**A** & **C**) and *Swarnaprabha* (**B** & **D**).

**Figure 2:**
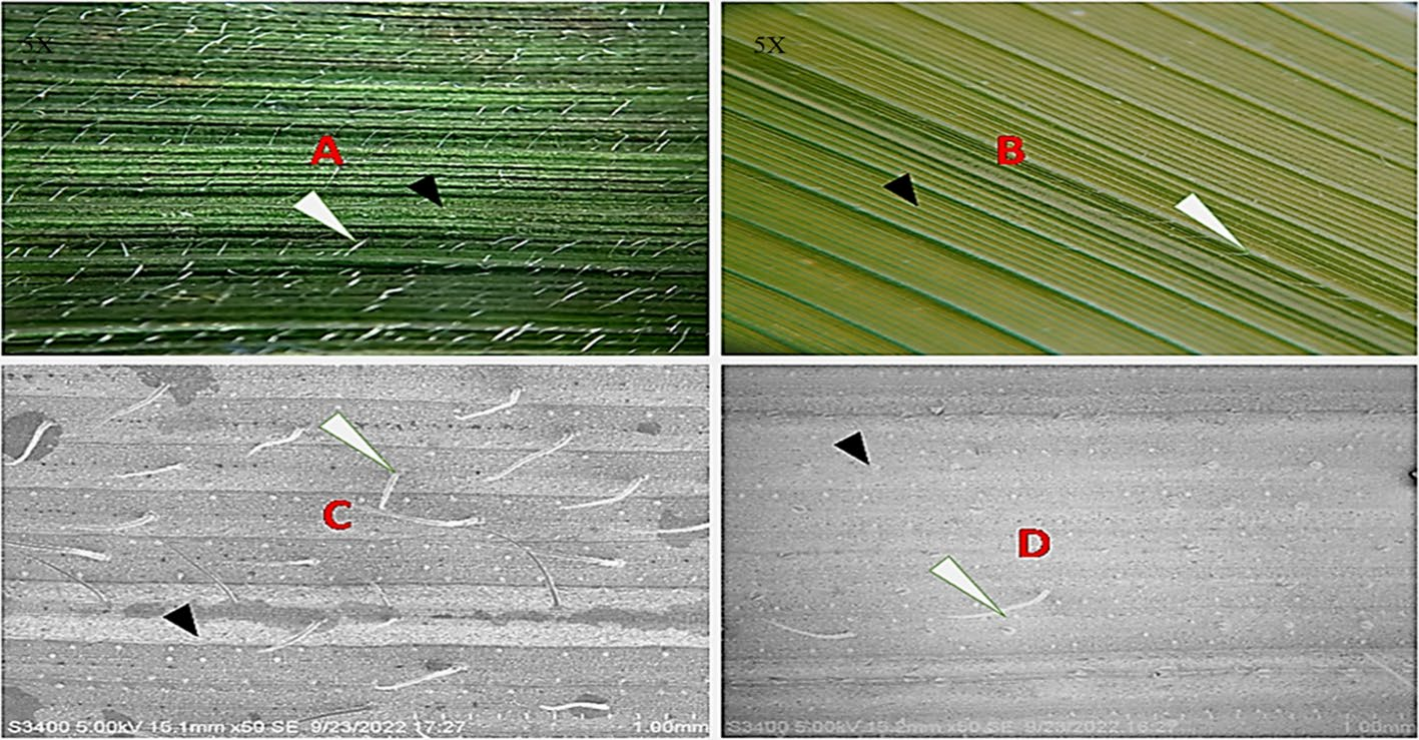
Microscopic photographs (**A** & **B**) and scanning electron microscope (SEM) views (**C** & **D**) of the adaxial surface of flag leaf of *Lalkain* (left panel) and *Swarnaprabha* (right panel) rice cultivars showing leaf pubescence.

Exposition to UV-B stress damaged the flag leaf of the sensitive cultivar Swarnaprabha with burning symptoms appearing on the leaf tip (Fig. 1B). The panicle size also reduced and most of the grains looked partially filled or unfilled (Fig. 1D). In contrast, the stress had a marginal impact on the resilient cultivar Lalkain; there was no leaf-burning and panicle size remained intact (Fig. 1A,C). The panicle grain number did not change, although some grains appeared blurry. The microscopic view of the flag leaf showed the presence of both macro and micro pubescent hair bristles on the leaf surface of both sensitive and insensitive cultivars; the number and size of the pubescent hairs (Trichomes) were lower in the former (Fig. 2B,D) compared to the latter (Fig. 2A,C). UV-B radiation depressed hair size most prominently in Swarnaprabha, but was not as effective in Lalkain.

#### Photosynthetic pigments of flag leaf

**Figure 3:**
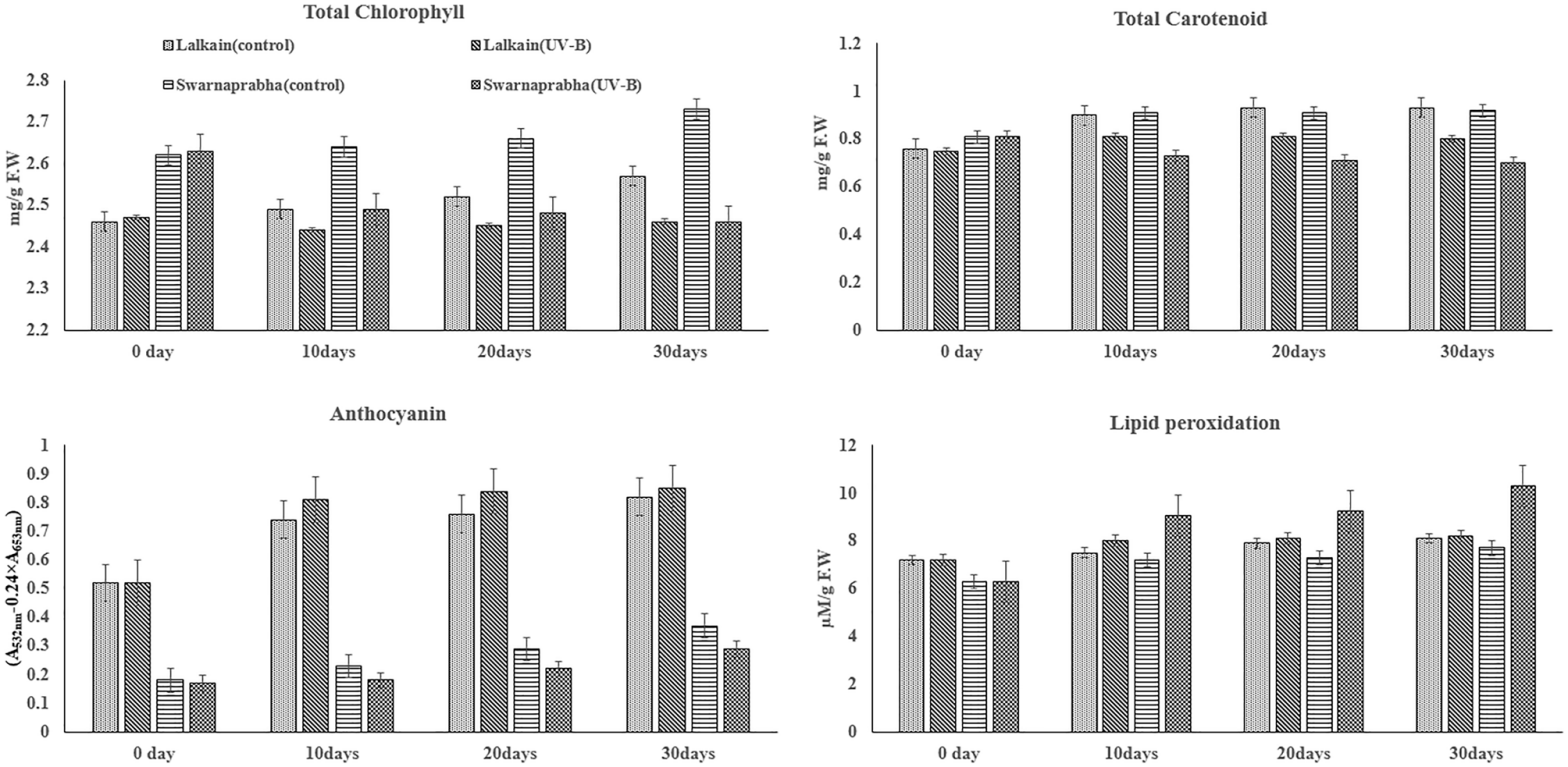
Effect of UV-B radiation on total chlorophylls, carotenoids, anthocyanin and lipid peroxidations of flag leaf of *Lalkain* and *Swarnaprabha* rice genotype on 0 (7 days before panicle emergence), 10, 20 and 30 days after treatment. Vertical bars at each observation point indicate mean ± standard deviation (n = 3) at P* < 0.05. The difference between control and UV-B treatment was statistically significant for total chlorophyll of flag leaf (t = 4.84***); carotenoid of flag leaf (t = 9.08***); anthocyanin of flag leaf (t = 4.67***); lipid peroxidation of flag leaf (t = 2.76*). (***P ≤ 0.001).

The total photosynthetic pigment contents, both chlorophylls and carotenoids in the flag leaf increased temporally in the control condition in both Lalkain and Swarnaprabha (Fig. 3). Exposition to UV-B stress depressed the pigment contents, more in the latter than in former. Lalkain had anthocyanin pigment contents nearly three times more than Swarnaprabha on all four occasions of sampling and it increased over time. UV-B stress marginally increased the pigment contents in Lalkain compared to the control. In Swarnaprabha, the stress reduced the pigment contents significantly. In the control plants of both cultivars’ lipid peroxidation escalated slowly over time. Exposure to UV-B stress enhanced the lipid peroxidation activity more in the sensitive cultivar Swarnaprabha than in the resistant cultivar Lalkain.

#### Photosynthetic parameters

Similar to the impact of pigment contents, UV-B stress also infringed upon the other photosynthetic parameters of the flag leaf (Table 2). The photosynthetic parameters like SPAD index, Fv/Fm, ETR, qP and NPQ were of similar values in the two cultivars Lalkain and Swarnaprabha. However, the other indicators for photosynthesis like Fm, PI abs, ABS/CS_0_ and ET_0_/CS_0_ were higher in the latter compared to the former under control conditions. Subject to UV-B stress, there was a significant decline in photosynthetic parameters like SPAD index, FV/Fm, ETR, Fm and qP in both cultivars, but the margin of fall was greater in Swarnaprabha than in Lalkain. The radiation treatment increased NPQ, PI abs, ABS/CS_0_ and ET_0_/CS_0_ indices in Lalkain, but reduced them in Swarnaprabha. Although grain number per plant was higher in Swarnaprabha than, in Lalkain control condition plants, some plants were sterile and appeared physically blurry under stress (Fig. 1).

**Table 2:** Photosynthetic parameters such as SPAD index; photochemical efficiency (Fv/Fm) of PSII; electron transport rate, ETR; photo-chemical quenching, qP; non-photochemical quenching, NPQ; minimal fluorescence, F_0_; maximal fluorescence, F_m_; performance index, PI abs; absorption flux per cross section, ABS/CS_0_; trapped energy flux per cross-section, ET_0_/CS_0_; percentage of sterility and fertile grain number of *Lalkain control (LKC)*, *Lalkain treatment (LKT)*, *Swarnaprabha control (SPC)* and *Swarnaprabha treatment (SPT)* at maturity stage after exposed to 30 days of UV-B treatment.  ± Values indicates SD values (n = 3). *P ≤ 0.05, **P ≤ 0.01

Name	SPAD index	Fv/Fm	ETR	qP	NPQ	F_0_	Fm	PI abs	ABS/CS_0_	ET_0_/CS_0_	% sterility	Fertile grain number
LKC	47.29 ± 0.57	0.58 ± 0.05	56.30 ± 4.85	0.66 ± 0.09	0.62 ± 0.05	93.66 ± 2.30	177.33 ± 47.54	1.56 ± 0.44	394.33 ± 11.93	158.33 ± 13.64	05.92 ± 0.21	208.00 ± 09.53
LKT	44.11 ± 0.57	0.45 ± 0.05	46.96 ± 4.32	0.54 ± 0.03	0.79 ± 0.08	87.66 ± 2.51	159.66 ± 47.68	2.69 ± 0.71	492.00 ± 28.47	214.86 ± 27.22	16.50 ± 0.71	178.33 ± 06.02
SPC	48.01 ± 0.45	0.64 ± 0.06	59.73 ± 2.37	0.65 ± 0.02	0.61 ± 0.01	84.33 ± 2.08	206.33 ± 32.12	2.70 ± 0.20	498.66 ± 13.86	226.61 ± 09.26	09.03 ± 0.43	317.00 ± 05.29
SPT	23.13 ± 0.63	0.36 ± 0.03	32.06 ± 4.31	0.46 ± 0.04	0.44 ± 0.03	69.33 ± 1.52	151.33 ± 11.93	2.16 ± 0.90	487.33 ± 39.50	191.98 ± 21.08	41.82 ± 1.80	171.00 ± 13.74
t-value between control and treatment (df = 5)	2.88*	2.99*	4.21**	4.94**	3.25*	4.61**	2.58*	0.67 NS	2.78*	2.89*	4.35**	3.35*

#### Fluorescence intensity spectra of flag leaf

The fluorescence intensity spectra of the flag leaf showed two peaks at 460 and 680 nm in both varieties (data not presented) exhibiting light energy utility regions of the leaf for photosynthesis. In the control condition, fluorescent intensity was very high at 460 and 680 nm for cultivar Swarnaprabha, which dwindled dramatically under exposure to UV-B stress. In comparison, fluorescent intensities under both the light wavelengths were lower in Lalkain, where UV-B stress had a marginal impact at 460 nm, but depressed it at 680 nm.

#### Phenolic compounds and flavonoid concentrations

**Figure 4:**
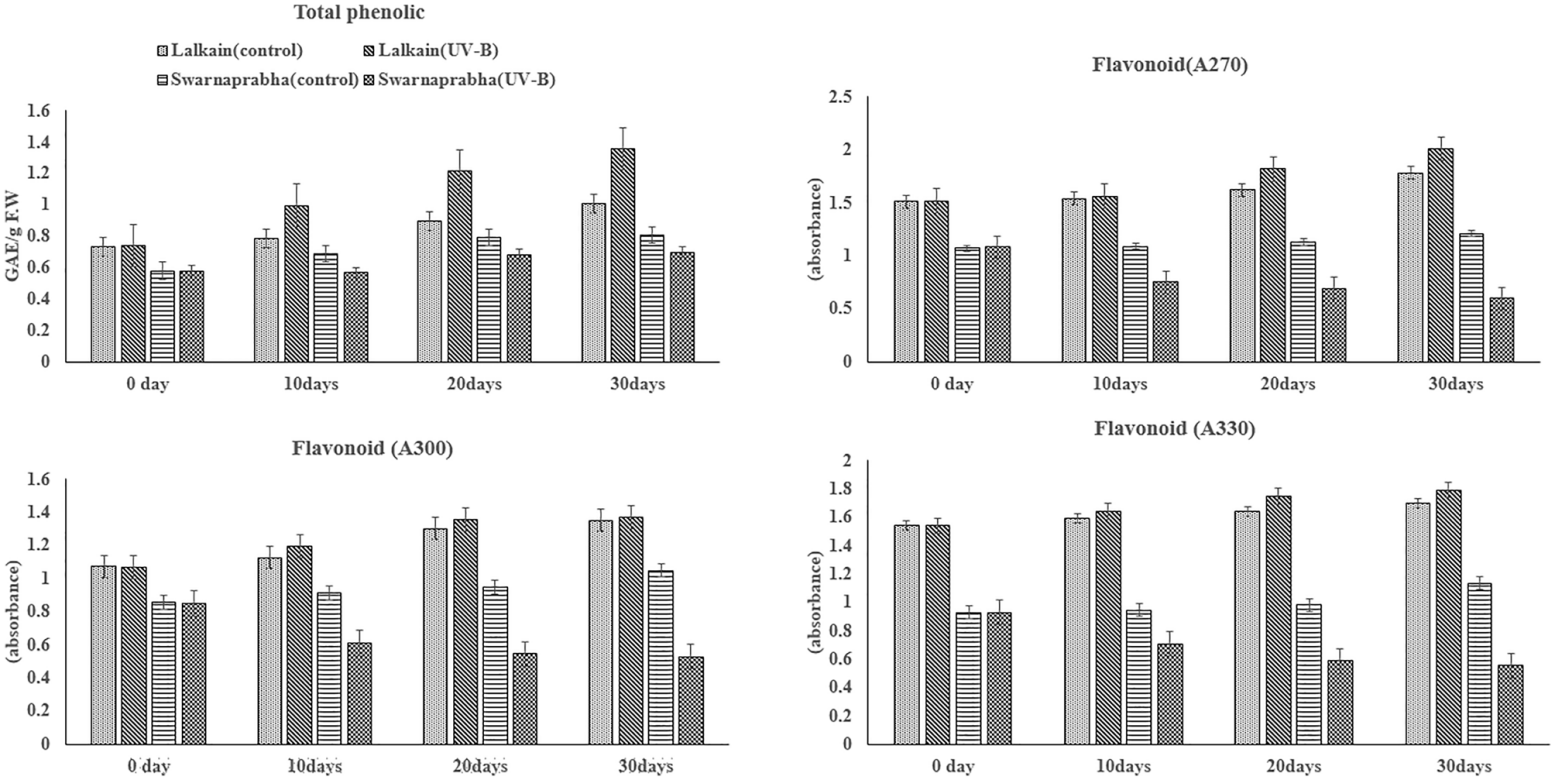
Effect of UV-B radiation on total phenolic contents and absorbance of total flavonoids at 270 nm, 300 nm and 330 nm of flag leaf of *Lalkain* and *Swarnaprabha* rice genotype after 0 (7 days before panicle emergence), 10, 20 and 30 days of after treatment. Vertical bars at each observation point indicate mean ± standard deviation (n = 3) at P* < 0.05. The difference between control and UV-B treatment was statistically significant for total phenolic contents (t = 2.77*); flavonoids at 270 nm of flag leaf (t = 1.90^NS^); flavonoids at 300 nm of flag leaf (t = 2.80**); flavonoids at 330 nm of flag leaf (t = 2.27*). (**P ≤ 0.01).

The total phenolic compounds in the flag leaf were higher in Lalkain than that of Swarnaprabha and the concentration increased over time irrespective of UV-B exposure (Fig. 4). In Lalkain, imposition of UV-B stress increased phenolic concentration, whereas the treatment depressed it in Swarnaprabha. The flavonoid contents of the flag leaf exhibited a pattern of response to the stress similar to the phenolic compounds. In the Lalkain leaf, the flavonoid concentration was higher than that of Swarnaprabha; the concentration increased temporally in Lalkain but decreased in Swarnaprabha.

#### Relative expression of flavonoid activating and UV-B signal transducer genes

**Figure 5:**
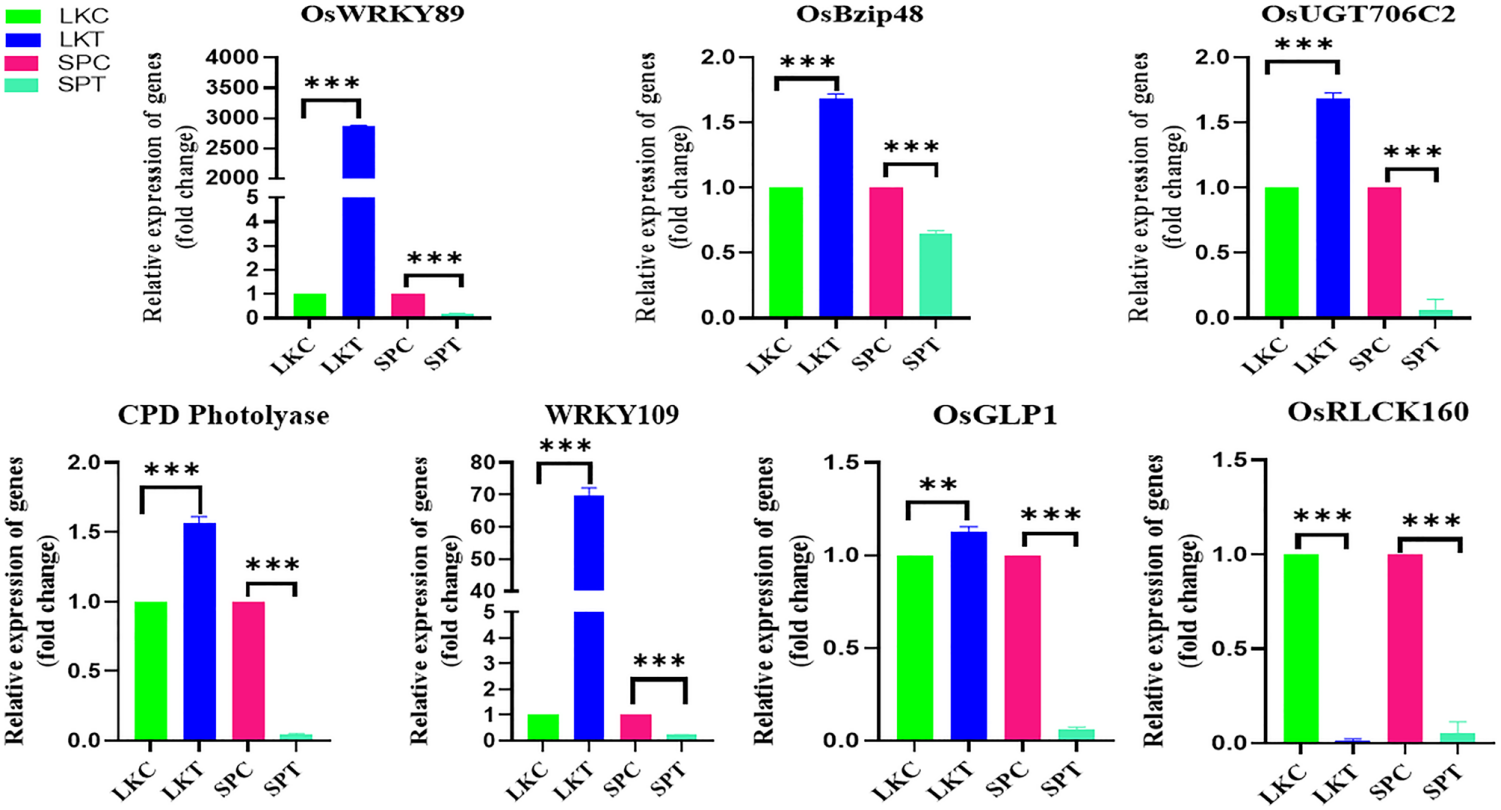
The effect of UV-B radiation on comparative expression analyses of flavonoid activating and UV-B signal transducer genes of *Lalkain* and *Swarnaprabha*. The CPD Photolyase gene has been effective in the correction of UV-B-induced DNA lesions in photo-reactivation. A positive interaction between receptor-like kinase OsRLCK160 and phosphorylated bZIP transcription factor OsbZIP48 is responsible for flavonoid pigment accumulation. OsGLP1 directly participates in acclimation to UV-B radiation. Down-regulation of OsGLP1 expression is responsible for the harmful effects of UV-B radiation. Upregulation of WRKY89 and WRKY109 genes confers UV-B resiliency.

Comparison of genes such as *OsWRKY 89*,* OsBzip 48*,* OsUGT706C2*,* CDP-Photolyase*,* WRKY109*,* OsGLP1* and *OsRLCK160* revealed identical relative expression in the flag leaf between Lalkain and Swarnaprabha under control conditions (Fig. 5). Imposition of UV-B stress increased expression of all the genes, except *OsRLCK 160* in Lalkain. Under stress, this gene expression was downregulated in both the cultivars in contrast to the response on the other genes. In the UV-B-sensitive Swarnaprabha, the expression of all seven genes studied was downregulated by the stress.

## Discussion

### Genotype screening

In our study, the response to UV-B stress among traditional rice cultivars was species-specific and accordingly, the cultivars were grouped into four distinct categories (Table 1). The phenotyping for UV-B sensitivity among indigenous genotypes at the reproductive growth stage was a novelty and not paralleled in the new and genetically modified high-yielding rice. So long, modern rice cultivars have retained a large introgression of beneficial traits like high grain yield, short stature for lodging resistance, high tillering, and photo insensitivity, based on genetic materials from China, Taiwan and Indonesia^18,19^ and disease resistance from wild rice^20^, but lack a source for UV-B resiliency. Also, the rate of rice yield increase based on genetic changes waning with the progress of time after the early success of the Green Revolution, high-yielding rice does not hold potential for future food security^11^ and the emergence of new environmental stresses can dampen the prospects further^21^. Because of the late emergence in rice domestication, high-yielding rice becomes incompatible with stress-prone environments, including UV-B stress, unless and otherwise, indigenous rice genotypes are phenotyped^22^ and sourced for novel genes. Against this background, our study screened 38 genotypes and phenotyped them for UV-B sensitivity with the help of several biomarkers inclusive of morphological and physiological features. The identity of one highly UV-B-resistant genotype like Lalkain and 19 other resistant cultivars (Table 1) was highly informative. The classification used in our study is synonymous with the segregation of *indica* and *japonica* rice seedlings that have been experimented with in the USA^17^. The authors classified 64 genotypes into four groups based on the impact of UV-B stress on morpho-physiological features. Similar to our study, the objective of the experiment was intended to help rice breeders achieve better yields.

### Physiological calibration of UV-B stress injuries

In our study, the magnitude of UV-B stress injuries on traditional rice cultivars was calibrated by following the effects of the stress on the flag leaf of the resistant cultivar Lalkain against the backdrop of the most sensitive cultivar Swarnaprabha. In traditional rice plants, grain development is sustained primarily on activity flag leaf in the supply of photosynthetic assimilates (Mohapatra et al.^23^). The pernicious effects of natural stress on flag leaf phytochemistry inducing photo-oxidative stress have been used as an index for survival (Kariali et al.^24^). Hence, we judge the sensitivity of rice plants exposed to UV-B radiation and their productivity based on the state of the flag leaf. Unlike our study conducted at the grain-filling stage, Faseela and Puthur^6^ screened popular rice cultivars for UV-B stress tolerance by analysis of growth, photosynthetic pigments and the rate of lipid peroxidation at the seedling stage. The sensitive seedlings were injured; there was growth reduction, and a decrease in total chlorophyll contents while lipid peroxidation increased. Our results corroborate these observations in traditional rice in the grain development stage, which is the most crucial period of economic yield and appropriate to the objective. Not only did the total chlorophyll contents decline but the stress also inhibited various photosynthetic parameters like SPAD, Fv/Fm, ETR and qP significantly (Fig. 3 and Table 2), which in turn increased grain sterility by restriction of photo-assimilate supply. According to Faseela and Puthur^6^, UV-B treatment damages PSI and PSII activity in seedlings of sensitive rice cultivars, inhibiting photochemical efficiency and promoting dissipated energy in heat or fluorescence. According to Bhattacharyya^4^, UV-B stress-induced photoinhibition of PSII increases non-photochemical quenching (NPQ), lipid peroxidation and protective accumulation of antioxidant compounds in the leaf. In our study, the stress reduced both photochemical and non-photochemical quenching in the sensitive cultivar Swarnaprabha (Table 2) owing to the loss of chlorophylls and flavonoid contents respectively (Figs. 3 and 4). In contrast, loss of chlorophyll contents and SPAD values was relatively minimal for the tolerant cultivar Lalkain, because photochemical quenching declined less and non-photochemical quenching increased under exposure to the stress. Attenuated response on photochemical quenching protected the cultivar to maintain the activity of PSII reaction centres and the stability of the primary quinone acceptor, while non-photochemical quenching de-excited UV-B induced excitation energy by thermal dissipation process^25^. Further, our study identified that fluorescent intensity, normally used as a scale for light (photons) emitted, was far lower in Swarnaprabha compared to Lalkain and being unable to do so, the former was more sensitive than the latter. Additionally, the absence of trichomes on the leaf surface for diffusion or reflection of UV-B radiation made Swarnaprabha vulnerable (Fig. 2)^26^. Therefore, leaf-burning symptoms appeared in Swarnaprabha while Lalkain remained protected (Fig. 1). Lalkain could gain this advantage for neutralisation of UV-B stress impact on leaf photosynthesis and minimising adverse responses on grain yield owing to the possession of higher levels of UV-B absorbing phenolic compounds and flavonoids (Fig. 4) and presence of pubescent hairs on the leaf surface (Fig. 2). The pubescent hairs can reflect UV-B radiation and constitute a shield against UV-B radiation^27^. Phenolics with flavonoid compounds work as non-enzymatic antioxidants for initiating DNA damage repair and preclude UV-B-induced oxidative stress injury^22,28^. Further, our study vindicated the diversity of phenolic concentration among Asian rice genotypes that can used for calibration of UV-B tolerance^1,29,30^**.**

### Genes controlling UV-B sensitivity

The proteomic response of rice leaves to UV-B stress was cultivars-specific (Sah et al.^31^) suggesting diversity in stress tolerance. Thus, it becomes highly pertinent to discover traits identifying differential expression patterns of genes controlling the stress effect. Jan et al.^22^ reported recently that there are several genes, the manipulation of which plays a crucial mediating role in allowing rice plants to acclimate and endure difficulties encountered by high UV-B radiation in their respective habitats through regulation of signalling pathways and gene expression^32^. In our study, the expression of CPD (Cyclobutane Pyridine Dimer) Photolyase was identical in both Swarnaprabha and Lalkain cultivars under control conditions. However, it overexpressed in the latter while dwindling in the former under UV-B treatment (Fig. 5). CPD is produced in the leaf chloroplast and mitochondrial DNA by UV-B radiation, and CPD photolyase repairs the damage^12^. The CPD photolyase gene has been effective in the correction of UV-B-induced DNA lesions in photo-reactivation, and it is a key determinant of stress sensitivity in rice^33^. Therefore, the overexpressed CPD photolyase was responsible for the greater resiliency of Lalkain to UV-B radiation^34,35^. In another facet of genetic regulation of UV-B resistance, Zhang et al.^13^ have identified a positive interaction between receptor-like kinase OsRLCK160 and phosphorylated bZIP transcription factor OsbZIP48 responsible for flavonoid pigment accumulation needed for filtration of harmful UV-B radiation on rice. In our study, OsRLCK160 expression was significantly downregulated by UV-B exposure in both sensitive and resistant rice cultivars, but OsbZip48 overexpressed only in the latter (Fig. 5). It is possible that overexpression of the transcription factor OsbZIP48 in conjunction with rice UDP-dependent glycosyl transferases (OsUGT706c2) in Lalkain improved UV-B tolerance, similar to the observation of Peng et al.^28^.

The WRKY group of transcription factors family is one of the largest involved with important and indispensable roles in normal plant development and abiotic stress response relating to drought, heat, cold, salinity and UV radiation^36,37^, but there is scant information in rice. Our endeavour identified significant upregulation of WRKY89 and WRKY109 genes in the UV-B resilient cultivar Lalkain subject to stress, but not in the sensitive cultivar Swarnaprabha (Fig. 5). Our study also identified the role of GLP proteins in UV-B resistance in rice. OsGLPs are primarily involved in lignin biosynthesis and deposition in rice to counter Cd and Cu toxicity^38^. However, its role in biotic and abiotic stress tolerance was emphasised in rice prominently^39,40^. More recently, it has been shown that OsGLP1 directly participates in acclimation to UV-B radiation; knock out of the gene using CRISPR/Cas9 resulted in a mutant rice plant (*glp1*) that exhibited UV-B dependent formation of lesion mimic in leaves and direct exposure to the stress resulted in decreased plant height and wider leaf angle^15^. Although injurious responses of UV-B stress on sensitive cultivar Swarnaprabha were not identical^15^, our study revealed down-regulation of OsGLP1 expression was responsible for the harmful effects (Fig. 5). Hence, expression of these genes could be forerunners for UV-B sensitivity and their transgression into sensitive cultivars could enhance grain yield under stress-prone environments.

## Conclusion

Our study screened 38 unimproved and immaculately clean rice landraces of western Odisha, India for UV-B sensitivity. UV-B stress infringed upon plant phenotypic growth by injuring the photosynthetic processes in sensitive cultivars. The resistant cultivars remained insensitive to UV-B radiation for phenolic compounds like anthocyanin and flavonoids in the leaf and maintained grain production. Relative expression of flavonoid activating and UV-B signal transducer genes was higher in the resilient cultivars.

## Materials and methods

### Plant material and experimental site

Thirty-eight traditional unimproved rice cultivars were collected and identified by PKS, the first author, from various farmers of western Odisha, India (Mr Narayan Naik, Basantpur, Mr Sudam Sahu, Katapali, Mr Kishor Kumar Kariali, Jamunadhipa). The genotypes were grown in cemented pots (330 × 330 × 260 mm) under open field conditions in the Botanical Garden of the School of Life Sciences, Sambalpur University in the Kharif season 2022. The genotypes were available in the School of Life Science, Sambalpur University with access to the public. The experimental design was a randomised block design with three replicates. The cemented pots were filled with a mixture of sandy loam soil and compost (10:1). Twenty-two-day-old seedlings grown in a nursery plot were transferred into concrete pots after following all prescribed agronomic standards. The surface water level was maintained at 5 ± 2 cm except one week after transplantation and before maturity. The photoperiod during the crop growth period receded from 12 h 18 min to 11 h 7 min. The mean, maximum, and minimum daily temperatures were 26.76 ± 3.96 °C, 32.37 ± 2.81 °C, and 21.15 ± 5.59 °C, respectively. The mean daily precipitation was 7.96 ± 5.60 mm. N, P_2_O_5_ and K_2_O were applied at 80: 40: 40 kg/ha respectively. All P_2_O_5_ was applied as basal at transplanting. N application was split into 25%, 50%, and 25% proportions, while K_2_O application was split into 50%, 25%, and 25% proportions. These splits were applied 5 days after transplanting, 25 days after transplanting (during the active tillering phase), and 45 days after transplanting (during the panicle initiation stage), respectively. The fertiliser sources used for the nutrients mentioned were Urea, DAP (Diammonium phosphate) and MOP (Muriate of Potassium).

### UV-B treatment

The plants were grown in open field conditions under natural sunlight. During the UV-B treatment period, the plants were exposed to supplemental UV-B radiation by an adjustable UV-B chamber. During UV-B treatment natural sunlight also came. Hence, the light condition was a combination of natural sunlight with supplementary UV-B radiation. UV-B tube (Philips TL 20W/01) emits only a very narrow waveband from the ‘B’ bandwidth of the UV spectrum (290 to 315 nm). This narrow waveband is between 305 and 315 nm and peaks (λmax) at 311 nm.

In the present situation the range of UV-B intensity comes from sunlight in the experimental habitat varies from 3.50 KJm^−2^d^−1^ to 7.23 KJm^−2^d^−1^ (Balasaraswathy et al.^41^) and that intensity did not remarkably damage the cultivars. But shortly under the climate change scenario, it may increase above it. For future perception, we used a supplementary UV-B dose, combining sunlight with a supplementary UV-B radiation of 14.4 KJm^−2^d^−1^. For uniformity of light intensity in our experiment, we provided a constant UV-B dose of 14.4 KJm^−2^d^−1^^42^ by minor adjustment in time of exposure according to variable natural conditions. UV-B intensity was measured with the PMA2106 UV-B sensor (Solar Light Co., USA) connected to the PMA 2200 radiometer (Solar Light Co., USA). The light intensity measured was between the surfaces of the UV-B tube (Philips TL 20W/01) and the canopy of the plants. UV-B lamps were turned on for 5 h per day from 9:00 am to 2:00 pm, which was the major photoperiod time.

Thirty-eight rice cultivars were subjected to UV-B radiation for 5 h per day (UVB_BE_14.4 KJm^−2^d^−1^) during the 7 days before panicle emergence. After 10, 20 and 30 days of supplementary UV-B treatments, morpho-physiological and biochemical tests of 38 rice cultivars were conducted. Following the preliminary tests conducted, two distinct rice cultivars, one sensitive and the other resistant, were chosen from the 38 rice cultivar stock for further investigation of the UV-B radiation effects on photosynthetic parameters, enzymatic activity, absorption-fluorescence mechanism, and various gene expressions controlling sensitivity features.

### Morphological parameters

Phenological observations were measured at maturity, such as plant height, flag leaf area, number of tillers, sterility percentage, and specific gravity of grains. The leaf surface area was computed by multiplying leaf length and maximum breadth by a constant factor of 0.69^43^.

$${\text{Leaf}}\;{\text{surface}}\;{\text{area }}\left( {{\text{cm}}^{{\text{2}}} } \right)\, = \,{\text{leaf}}\;{\text{length}}\, \times \,{\text{width}}\, \times \,0.{\text{69}}$$

### Biochemical and biomolecular studies

The flag leaf of the plants was sampled for various biochemical and bimolecular studies. This is the organ physiologically most active in the rice plants for primary production^23^.

### Chlorophyll and carotenoids estimation

The total chlorophyll contents were estimated using the Arnon^44^ method, while the carotenoid concentrations were measured using the procedure of Macalacham and Zalik^45^. 1 g of fresh leaf sample was homogenised with pre-chilled 80% aqueous acetone before centrifugation at 5000 rpm for 10 min. The supernatant was collected in a 100 mL volumetric flask. The residue was dissolved in 80% acetone, centrifuged for another 10 min, and the supernatant was pooled. The supernatant volume was made up to mark (100 mL) in a volumetric flask with distilled water, and absorbance was measured at 480, 510, 645, and 663 nm wavelengths. The concentrations of chlorophylls and carotenoids were determined using the following formula.

$$\text{Total chlorophyll }(\text{mg}/\text{g})=\frac{20.2\left(\text{A}645\right)+8.02(\text{A}663)\times \text{V}}{1000}\times \text{W}$$

$$\text{Carotenoid}(\text{mg}/\text{g})=\frac{7.6(\text{A}480)-1.49(\text{A}510)\times \text{V}}{1000}\times \text{W}$$

### Flavonoids concentration

The flavonoids were quantitatively estimated using the Krizek et al.^46^ approach. 0.1 g of fresh leaf tissue was extracted in a 15 mL glass centrifuge tube containing 10 mL of ethyl alcohol: acetic acid (99:1 v/v). The samples were gently heated for 10 min in a water bath at 80 °C, and the volume was adjusted to 10 mL. The absorbance was measured at 270, 300, and 330 nm wavelengths using a UV-Vis Spectrophotometer (Shimadzu, Model No. 05659).

### Anthocyanins concentration

Murray and Hackett's^47^ technique was used to estimate the anthocyanin concentration. 0.1 g of fresh leaf sample was extracted in a 15 mL glass centrifuge tube containing 10 mL of acidified methanol (methanol: HCl 99:1; v:v) and stored in the dark at room temperature overnight. The volume was adjusted to 10 mL by adding acidified methanol before the absorbance was measured at 532 nm and 653 nm. The anthocyanin absorbance was estimated by subtracting the non-specific absorption by chlorophyll that is, by subtracting 24% of the chlorophyll absorbance at A653 from A532.

$${\text{Actual}}\;{\text{absorbance}}\;{\text{by}}\;{\text{Anthocyanin}} = ({\text{A}}532 - 0.24 \times {\text{A}}635)$$

### Lipid peroxidation

Lipid peroxidation was assessed using Heath and Packer's^48^ technique. 0.2 g of fresh leaf tissue was weighed, homogenized in 5 mL of 5% trichloroacetic acid, and centrifuged at 12,000 rpm for 15 min. 2 mL of the supernatant was combined with an equal volume of 0.5% thiobarbituric acid in 20% trichloroacetic acid, and the mixture was heated at 95 °C for 24 min. After cooling, the mixture was centrifuged at 3000 rpm for 5 min, and the supernatant’s absorbance was measured at 532 nm and 600 nm using a UV-Vis Spectrophotometer (Shimadzu, Model No. 05659). The MDA content was determined using the extinction coefficient (ε) of 155 mM^−1^ cm^−1^.

$${\text{MDA(}}\upmu {\text{M/g)}} = \frac{{({\text{A}}532 - {\text{A}}600) \times {\text{V}}}}{{\upvarepsilon \times {\text{W}}}}$$

(A=absorbance, V= volume of supernatant, W= weight of sample, ε = extinction coefficient).

### Total phenolic contents

The total phenolic contents of the extracts were measured using the Folin-Ciocalteu reagent^49^. Sample and standard readings were made using a spectrophotometer (Shimadzu, Model No. 05659) at 765 nm against the reagent blank. The test sample (0.2 mL) was combined with 0.6 mL water and 0.2 mL Folin-Ciocalteu phenol reagent (1:1). After 5 min, 1 mL of saturated sodium carbonate solution (8% w/v in water) was added to the combination, and the volume was increased to 3 mL with distilled water. The reaction was left in the dark for 30 min, and after centrifugation, the absorbance of blue colour from several samples was measured at 765 nm. The phenolic content was estimated as gallic acid equivalents (GAE) per gram of fresh plant material using a gallic acid reference curve.

### SPAD index

The SPAD indices of leaf samples were measured on completely expanded leaves of various plants in three repetitions using a SPAD 502 chlorophyll meter (Konica Minolta Sensing, Inc., Osaka, Japan), and the SPAD values were determined while the transmitted light intensity was at 650 nm.

### Chlorophyll fluorescence

Fluorescence parameters were measured with a portable chlorophyll fluorometer (JUNIOR-PALM, WALZ, Germany). In 20-min dark-adapted leaves, various chlorophyll fluorescence parameters were evaluated, including minimal fluorescence (Fo), maximal fluorescence (Fm), maximum photochemical efficiency of PSII (Fv/Fm), and electron transport rate (ETR). The non-photochemical quenching (NPQ) and photochemical quenching coefficient (qP) were also calculated^50^.

### Fluorescence spectra

Fluorescence spectra were measured using a Spectrofluorimeter (Shimadzu, Model No. RF5001PC) with fresh leaf tissue's methanolic extracts (aqueous 80%). Leaf extracts were excited with a wavelength of 281 nm to produce fluorescence. Fluorescence spectra were obtained between 350 and 700 nm using a Spectrofluorimeter (Shimadzu, Model No. RF5001PC) equipped with a red-sensitive photo-multiplier R928 and a 150W Xenon lamp as the excitation source. The laser had a frequency of 10 Hz and an integration duration of 1 s for each fluorescence-emission spectrum.

### Scanning electron microscopy (SEM)

Trichomes on the adaxial surface of leaves were examined using SEM (Hitachi-s3400) with a resolution of 15.1 mm × 50SE and an area of 1 mm square.

### Quantitative real-time PCR (qRT-PCR) for comparative expression analyses of flavonoid activating and UV-B signal transducer genes

Total RNA was extracted from the flag leaves of both rice genotypes using Trizol reagent (Invitrogen). The extract was processed with DNase I (Promega) to remove genomic DNA contamination, which was then validated by PCR using 20 ng RNA as the template and Actin as the target gene. The genomic DNA-free RNA obtained as described above was reverse-transcribed using the QuantiTect Reverse Transcription Kit (Thermo Fisher Scientific). The generated cDNA was used as a template for qRT-PCR analysis using QuantiFast SYBR Green PCR Kit (Thermo Fisher Scientific) and Real-Time PCR equipment (Applied Biosystem 7500 Fast). Each selected gene was amplified with gene-specific primers generated by Primer Blast (NCBI). 18S rRNA was used as a reference control. The fold changes in gene expression were determined using 2ΔΔCt^51^. The reported data was the mean of three replicates. The primer sequences (forward and reverse) of various genes are provided in Table 3.

**Table 3:** Primer sequences for comparative expression analyses of flavonoid activating and UV-B signal transducer genes.

Genes	Forward primer	Reverse primer
OsRLCK160	5’-CAAGCATGAAAACCTCGTCA-3’	3’-GGGCGAATTTCCTCATGTAA-5’
OsbZIP48	5’-GTAGTGTTCGGGTTGCAGGT-3’	3’-TCCAGCTCACTCATGTACGC-5’
OsUGT706C2	5’-TCTTGGAGAGGACGAAGCAT-3’	3’-ATCTCCTCCACCATGAGCAC-5’
WRKY109	5’-TCAGTCCAAGAACAGGCACA-3’	3’-AAGCTGCACTCTTGGACCAT-5’
OsWRKY89	5’-ACCAAAAGGCTCTCATGGTG-3’	3’-CAAGTTGTGGTTGTCCATGC-5’
OsGLP1	5’-GCCTCCAGATCACCGACTAC-3’	3’-CAACTCGACCGGAGAGAGAC-5
CDP Photolyase	5’-CCGTCGATGCTTTCTTGGAGG-3’	3’-CATCTCCAACTGCGATGCATTCCA-5’

### Statistical analysis

All statistical analyses were performed using the Microsoft Excel 2013 computer programme.

### Compliance of the IUCN Policy Statement

Experimental research and field studies on plants (either cultivated or wild), including the collection of plant material, comply with relevant institutional, national, and international guidelines and legislation. The authors have complied IUCN Policy Statement on Research Involving Species at Risk of Extinction and the Convention on the Trade in Endangered Species of Wild Fauna and Flora.

## Data Availability

The datasets used and/or analysed during the current study are available from the corresponding author upon reasonable request.
